# Improved construction and applicability analysis of stomatal conductance model for cotton under drip irrigation in Northern Xinjiang

**DOI:** 10.3389/fpls.2026.1827847

**Published:** 2026-05-11

**Authors:** Wenting Qi, Ning Meng, Qianyu Cheng, Xiaopeng Ma, Weijie Wang, Jiandong Wang, Chuanjuan Wang, Hang Wang

**Affiliations:** 1 Institute of Environment and Sustainable Development in Agriculture, Chinese Academy of Agricultural Sciences, Beijing, China; 2 Western Agricultural Research Center, Chinese Academy of Agricultural Sciences, Changji, China; 3 State Key Laboratory of Efficient Utilization of Agricultural Water Resources, China Agricultural University/Chinese Academy of Agricultural Sciences (CAU/CAAS), Beijing, China; 4 School of Water Resources and Hydropower, Hebei University of Engineering, Handan, Hebei, China; 5 Institute of Soil Fertilizer and Agricultural Water Saving, Xinjiang Academy of Agricultural Sciences, Urumqi, China; 6 Linshu County Bureau of Agriculture and Rural Affairs, Linshu, Shandong, China

**Keywords:** leaf-air temperature difference, moisture response function, stomatal conductance model, stomatal conductance models, temperature response function

## Abstract

**Introduction:**

Traditional plant stomatal conductance (g_sw_) models have limited applicability. An urgent need exists to develop a g_sw_ model suitable for cotton grown under drip irrigation in Changji, Xinjiang, and to investigate the mechanisms underlying the effects of irrigation variations on leaf g_sw_.

**Method:**

This 2-year field study selected three g_sw_ models (Ball–Woodrow–Berry [BWB], Ball–Berry–Leuning [BBL], and unified stomatal optimization [USO]) and three optimization factors (water potential response function f[θ]), leaf temperature difference [ΔT], and temperature response function f[T]) to optimize, refine, and validate the most suitable g_sw_ model for cotton grown under plastic film drip irrigation.

**Results:**

Results revealed that the USO model exhibited the highest simulation accuracy, followed by the BBL and BWB models. Among the three models, the USO model incorporating ΔT, f(θ), and f(T) was the most accurate improved model. Compared to the BBL and BWB models, its 2-year *R*² values increased by 3.77%–49.75% and 1.94%–55.98%, respectively, 0.35%–38.10%, and 8.99%–45.21%, respectively. Furthermore, treatments with higher lower irrigation limits (F2B2 and F2B3) exhibited superior simulation accuracy compared to other irrigation deficit treatments, making them more suitable for the application of the improved model.

**Discussion:**

Overall, accurate estimation of g_sw_ requires the model to account for f(θ), ΔT, and f(T). Under conditions where soil moisture is maintained at 70% of field capacity, this improved model shows more precise simulation results. For cotton in the study region, the USO model incorporating ΔT&f(θ)&f(T) is recommended for simulating g_sw_.

## Highlights

- USO model accuracy is higher than those of the BBL and BWB models.
- Stomatal conductance model simulates mild stress (F2B2, F2B3) with higher accuracy.
- Improved USO-ΔT&f(θ)&f(T) model simulates stomatal conductance in Northern Xinjiang.

## 1 Introduction

In agricultural ecosystems, energy exchange and water vapor transport at the vegetation–atmosphere interface directly determines water use efficiency and crop productivity in farmlands. Dynamic changes in plant stomata, which are the key site regulating material exchange between leaves and the atmosphere, profoundly influence the interaction between the land surface and the atmosphere (Singh and Raja Reddy, 2011). Stomata serve as the primary pathway for carbon dioxide (CO2) uptake and water vapor release. Changes in their aperture directly affect net photosynthetic rate (A), transpiration, and water use efficiency (Campos et al., 2014). Therefore, accurate simulation of stomatal conductance (g_sw_) is crucial for elucidating crop physiological and ecological processes and predicting yield formation.

In recent years, as research on stomatal regulation mechanisms has deepened, various gsw models have been developed and widely applied across different crops (Wang et al., 2018). The commonly used models are primarily classified into three categories: empirical, semi-empirical, and mechanistically optimized. Empirical models, such as Jarvis, express stomatal response through the superposition of the product terms of environmental factors; however, they do not consider the interactions between factors, assuming that each environmental factor is independent (Jarvis, 1976). The Ball–Woodrow–Berry (BWB) semi-empirical model establishes a relationship between gsw and A as well as environmental humidity; however, simulation errors are significant at low CO2 concentrations, particularly near the CO2 compensation point (Ball et al., 1987; Jarvis, 1976). Leuning (1995) replaced relative humidity (h_s_) with vapor pressure deficit to propose the Ball–Berry–Leuning (BBL), thereby improving the plausibility of environmental responses. Mechanistic models, such as the unified stomatal optimization (USO) and Medlyn models, are based on the theory of optimal stomatal behavior, assuming that stomatal behavior aims to maximize carbon assimilation and minimize water loss (Medlyn et al., 2012). Among these models, the USO model, which exhibits strong mechanistic capability and universal applicability, features simple parameters and can be easily coupled with photosynthetic models. However, its response to abrupt changes in atmospheric CO2 concentration remains limited. Overall, existing gsw models have their inherent advantages; however, under the influence of regional climate and water stress, their simulation accuracy can be further improved through targeted optimization based on regional environmental characteristics.

Water stress significantly alters the water and heat transport characteristics of the soil–plant-atmosphere continuum, thereby influencing leaf photosynthetic physiological processes (Li et al., 2022); g_sw_ is highly sensitive to soil moisture conditions, and neglecting water stress often leads to an overestimation of g_sw_ (Egea et al., 2011; Keenan et al., 2010). Stomatal behavior is essentially the result of the combined effects of air dryness, soil water supply capacity, and crop physiological characteristics (Wang et al., 2018). From a physiological perspective, temperature directly regulates stomatal movement by influencing enzyme activity, membrane stability, and the rates of photosynthetic biochemical reactions. Therefore, a temperature response function (f[T]) can more accurately reflect the response of stomata to thermal conditions (Oliver et al., 2022). Soil moisture status determines root water uptake capacity and the degree of water deficit in plants. A soil moisture response function (f[θ]) can quantitatively characterize the constraining effect of water stress on stomatal aperture (Ji et al., 2017). The leaf–air temperature difference (ΔT) comprehensively reflects the balance between plant transpiration and atmospheric evapotranspiration demand, serving as a sensitive indicator of crop water stress (Fang et al., 2023). Incorporating it as a coupling factor helps improve the simulation stability of models under fluctuating water conditions. Peng et al. (2009) enhanced simulation performance under water-saving irrigation conditions by introducing ΔT to characterize water deficit. Li et al. (2024) enhanced model adaptability to temperature fluctuations by coupling f(T). Although single-factor improvements have yielded some results, the synergistic mechanisms of water stress, ΔT, and temperature response remain unclear. Moreover, studies on multi-factor coupling improvements are relatively limited. The applicability of g_sw_ models needs to be improved, particularly in arid regions with limited irrigation and frequent water fluctuations. Therefore, the systematic development of a multi-factor model incorporating water response functions, leaf temperature differences, and temperature response functions is of significant theoretical importance for enhancing the accuracy of g_sw_ simulation in arid regions.

Water scarcity has become a key bottleneck constraining the sustainable production of cotton in Xinjiang (Mao et al., 2024). The Changji region widely adopts a quota irrigation system, and cotton plants often experience periodic water stress during their growth period. Consequently, traditional gsw models struggle to accurately reflect stomatal dynamics under actual field conditions because they do not fully account for multi-factor synergistic constraints. To overcome the limitations of single-factor improvements, this study integrated f[θ], ΔT, and f[T] to construct a multi-factor g_sw_ model suitable for the cotton cultivated in Changji, Xinjiang. This approach not only enhances the reliability of stomatal behavior simulation but also provides scientific support for optimizing irrigation regimes and improving water use efficiency. This 2-year field study used the BWB, BBL, and USO models as a foundation to systematically achieve model improvements and evaluate their applicability. The specific objectives were as follows: (1) to systematically evaluate the simulation performance of the BWB, BBL, and USO models for the cotton cultivated in the study region and rank them; (2) to introduce f[θ], ΔT, and f[T] to construct single-factor and multi-factor improved models, thereby enhancing the accuracy of g_sw_ simulation; and (3) to assess the applicability of the improved models under different irrigation gradient scenarios and clarify their applicability scope. Compared with previous studies that have primarily focused on single-factor improvements, this study integrates water stress, leaf temperature differences, and temperature response into a unified framework, elucidating the mechanisms underlying the multi-factor synergistic regulation of stomatal behavior and addressing the issue of insufficient adaptability of traditional models under limited irrigation conditions. The findings provide methodological references for further investigation into the mechanisms of stomatal regulation in cotton in arid regions and offer model tools and theoretical foundations for precise irrigation decision-making and efficient water management.

## 2 Materials and methods

### 2.1 Experimental site

This study was performed at the Changji Comprehensive Experimental Base of the Western Agricultural Research Center, Chinese Academy of Agricultural Sciences (44°15'N, 87°19'E; elevation, 470 m). This region experiences a continental arid climate, characterized by an average annual precipitation of 170 mm, evaporation of 1,787 mm, a mean temperature of 6.6 °C, sunshine duration of 2,833 hours, and a frost-free period of ~175 days. The physical properties of the soil in this region are presented in Appendix **Table 1**.

**Table 1:** Arrangement of irrigation treatments.

Treatment		Seedling period		Squaring period		Flowering and boll setting period		Boll opening period	
		Lower limit for irrigation %FC	Upper limit for irrigation/%FC	Lower limit for irrigation %FC	Upper limit for irrigation/%FC	Lower limit for irrigation %FC	Upper limit for irrigation/%FC	Lower limit for irrigation %FC	Upper limit for irrigation/%FC
F1	F1B1	60	100	60	85	50	90	No irrigation	
	F1B2	60	100	60	85	60	90		
	F1B3	60	100	60	85	70	90		
F2	F2B1	60	100	70	85	50	90		
	F2B2	60	100	70	85	60	90		
	F2B3	60	100	70	85	70	90		
CK		Local conventional drip irrigation system for large fields							

FC indicates Field Capacity. F1 represents a minimum irrigation level of 60% field capacity (FC) during the budding stage, F2 represents a minimum irrigation level of 70% field capacity (FC) during the budding stage, F represents the sum of F1 and F2.

### 2.2 Field management and experimental design

**Figure 1:**
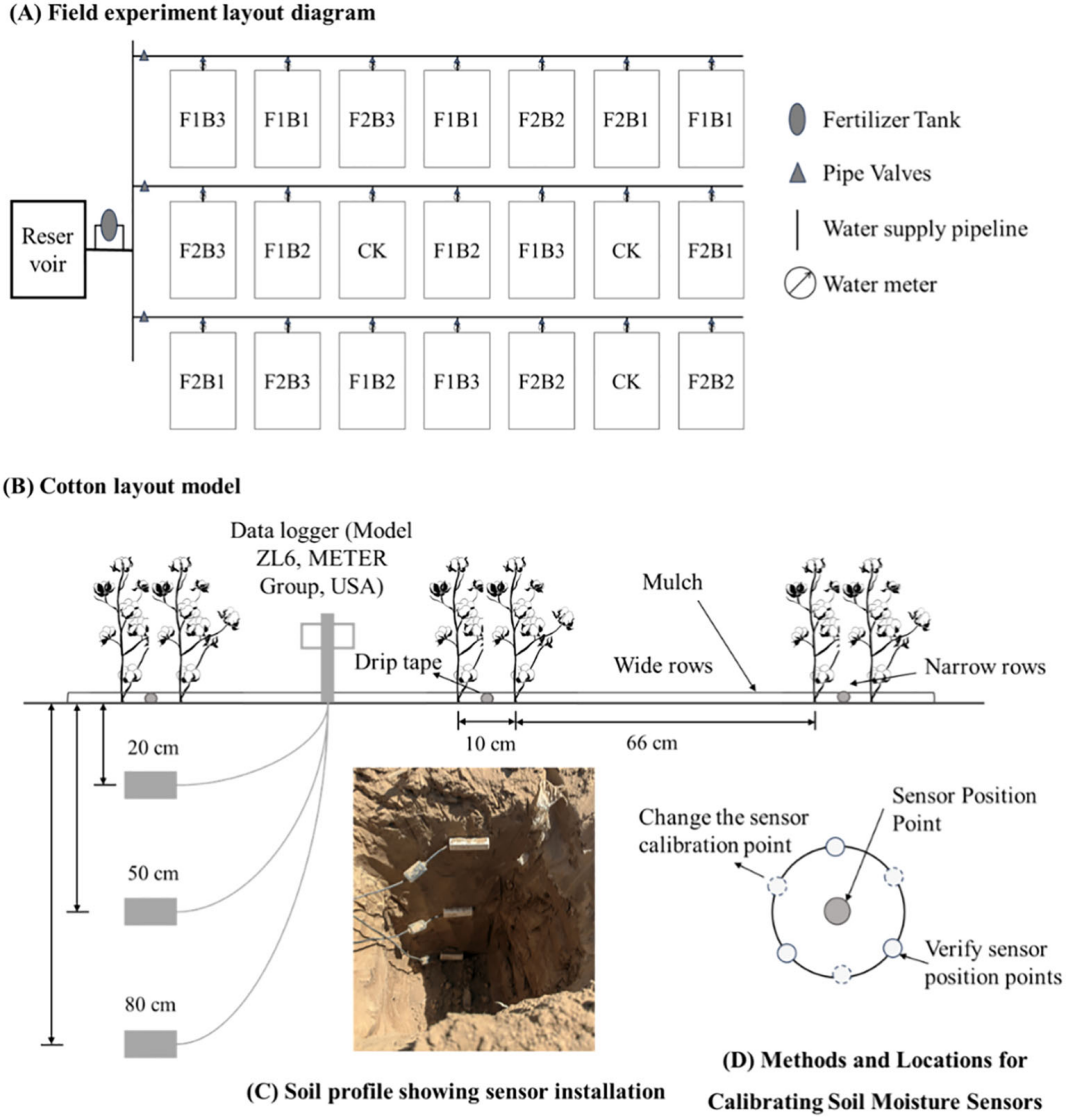
Cotton cultivation patterns.

This 2-year field study was performed from April 2023 to October 2024 using the cotton cultivar Cotton Institute 113. The field experiment was performed using drip irrigation under plastic mulch, with a wide–narrow row planting pattern consisting of one mulch strip, three drip tapes, and six rows. Row spacing was maintained at (66 + 10) cm, plant spacing was maintained at 10 cm, and planting density was set at 300,000 plants·hm^−2^ (**Figure 1B**). Patch-type drip tapes with a rated flow rate of 2.4 L·h^−1^ and a drip emitter spacing of 25 cm were used. Cotton consumes >70% of its total water requirements during the flowering and boll setting stages (Qi et al., 2024), making these stages critical for water consumption optimization and regulation. Therefore, we implemented different upper and lower limits of irrigation levels primarily during the budding and flowering stages while maintaining the same upper and lower limits during other growth stages. The experimental design is summarized in **Tables 1**, **2**. Each treatment was performed in three replicates, resulting in 21 plots. Each plot measured 4.5 m × 10 m, and the plots were randomly arranged (**Figure 1A**). The types and application rates of fertilizers used during the entire growth period of cotton were as follows: basal fertilizer, diammonium phosphate at 375 kg·hm^−2^; topdressing, urea at 600 kg·hm^−2^, potassium sulfate at 375 kg·hm^−2^, and monoammonium phosphate at 400 kg·hm^−2^ (the types, timing, and total amounts of topdressing for the control (CK) group are shown in **Table 2**). All other agronomic management practices (topping, pesticide application, and weed control) were consistent with local management practices for cultivation under drip irrigation (CK).

**Table 2:** Irrigation amount and types, timing and total amounts of topdressing in the CK.

Date		Irrigation amount/mm	Urea/(kg·hm^−2^)	Monammonium phosphate/(kg·hm^−2^)	Potassium sulfate/(kg·hm^−2^)
2023-04-29	2024-04-27	25	——	——	——
2023-06-05	2024-05-31	40	——	——	——
2023-06-20	2024-06-15	40	45	30	60
2023-06-30	2024-06-25	40	45	30	60
2023-07-10	2024-07-05	60	90	60	45
2023-07-20	2024-07-15	60	90	60	45
2023-07-30	2024-07-25	60	90	60	45
2023-08-10	2024-08-05	60	90	60	45
2023-08-20	2024-08-15	60	90	60	45
2023-08-30	2024-08-25	60	60	40	30
Total		505	600	400	375

The irrigation quotas for each treatment were determined using the moisture content difference method Equation 1, with soil moisture content (0–50-cm) was measured using a ZL6 monitoring system (TEROS12, METER Company, Pullman, WA, USA).

(1) $$M=1000\times P_{t}\times H\times \gamma \times (\theta_{\text{before}}-\theta_{\text{after}})/\eta$$

where M is the amount of irrigation water, mm; *θ*_before_ is the 0–50-cm soil water content (i.e., the average water content of the 20-cm and 50-cm soil layers) before irrigation, %; θ_after_ is the 0–50-cm soil water content after irrigation (**Table 2**; to determine the upper limit of the irrigation, seedling, squaring, and flowering and boll setting periods of the upper limit of the irrigation were 100% FC, 80%–85% FC, and 90%–95% FC), %; γ is the ratio of soil capacity to water capacity; H is the depth of the soil wetting layer, m (0.4 m in the seedling and squaring period, 0.6 m in the flowering and boll setting period); P_t_ is the wetting ratio, which is 0.6 (GB/T 50485-2020); η is the coefficient of irrigation water utilization, which was considered to be 0.95.

### 2.3 Observational indicators and measurement methods

#### 2.3.1 Soil moisture content

Data were systematically collected using a ZL6 monitoring system (TEROS12, METER, USA), installed on May 4, 2023, and May 1, 2024, and retrieved on September 19, 2023, and September 15, 2024. Sensors were vertically installed at depths of 20, 50, and 80 cm within the cotton root zone (**Figure 1C**). Data were recorded every 30 minutes and downloaded daily at 20:00 Beijing time using a data cable. The daily averages of all recorded values were used as the respective soil temperature, moisture content, and electrical conductivity data for each day.

To ensure the accuracy and reliability of the monitoring data, *in-situ* field calibration of soil moisture content was performed during the monitoring period using the oven-drying method. Calibrated soil samples were collected during the seedling (May 15, 2023, and May 15, 2024), squaring (June 15, 2023, and June 15, 2024), flowering and boll–setting period (July 15, 2023, and July 15, 2024). The samples were collected within a 10-cm radius around each sensor probe installation site. All sampling points were located at the same horizontal position and soil depth as the corresponding probes, ensuring consistent and adjacent spatial locations. To minimize the influence of soil spatial heterogeneity, three-point composite sampling was performed around each probe. This approach ensured that the collected soil samples accurately represented the actual soil conditions monitored by the sensors (**Figure 1D**). The samples were weighed immediately, oven-dried at 105 °C until a constant weight was achieved, and reweighed to calculate moisture content.

#### 2.3.2 Photosynthetic physiological indicators

A YaXin photosynthesis meter (Beijing Yaxinliyi Science and Technology Co., Ltd, Beijing, China) was used to measure daily photosynthetic indicators, including leaf temperature (*T_L_*), relative humidity (*h_s_*), net photosynthetic rate (A), stomatal conductance (*g_sw_*), intercellular carbon dioxide concentration (*C_i_*) and the CO_2_ concentration on the leaf surface (*C_s_*). Measurements began when the cotton entered the seedling stage. Each plot was replicated three times, with measurements performed every 5 d between 12:00 and 14:00 (GMT + 8) on clear, cloudless days. Sampling points were selected on the third leaf from the top, where the leaf was fully expanded, and avoided the main stem vein (Qi et al., 2024).

#### 2.3.3 Meteorological indicators

The Tianqi intelligent ecological station (Oriental Smart Sense, Zhejiang, China) was installed at the center of the test area to measure meteorological data, including daily solar radiation (S, MJ/m²), relative humidity (*RH*, %), air temperature (*T_a_*, °C), etc. Meteorological data were automatically uploaded once per hour and could be downloaded at any time. The vapor pressure deficit was calculated using Equations 2–5:

(2) VPD=SVP−AVP

(3) $$SVP=0.6112\times e^{\frac{16.67\times T_{a}}{T_{a}+243.5}}$$

(4) $$AVP=0.6112\times e^{\frac{16.67\times T_{dew}}{T_{dew}+243.5}}$$

(5) $$T_{dew}=T_{a}-(\frac{100-RH}{5})$$

Where *SVP* is the saturated vapor pressure, kPa; *AVP* is the actual vapor pressure, kPa; *T_dew_* is the dew point temperature, °C; *T*_a_ is the air temperature measured by the weather station, °C; *RH* is the relative humidity measured by the weather station, %.

### 2.4 Model description

Mulching and soil moisture conditions can alter soil temperature and humidity, thereby influencing leaf photosynthesis (Li et al., 2022). The present study employed three g_sw_-models (i.e., BWB, BBL, and USO), with modifications made to evaluate their applicability.

#### 2.4.1 Ball-Woodrow-Berry model

Ball et al. (1987) proposed that stomata can maintain stable intercellular CO_2_ concentrations. When humidity and CO_2_ are constant, *g*_sw_ and A exhibit a linear relationship. The BWB model is expressed as follows Equation 6:

(6) $$g_{SW}=m\frac{Ah_{s}}{C_{s}}+g_{0}$$

Where *g_sw_* is the stomatal conductance, mol·m^−2^·s^−1^; *C_s_* is the CO_2_ concentration on the leaf surface, μmol·mol^−1^; A is the net photosynthetic rate, μmol·m^−2^·s^−1^; *h_s_* is the air relative humidity on the leaf surface, %; m is an empirical coefficient, and g_0_ is the stomatal conductance value at the light compensation point. Both m and g_0_ are the fitting parameters.

#### 2.4.2 Ball-Berry-Leuning model

The relationship between *g_sw_* and A varies under different moisture conditions (Damour et al., 2010). Leuning replaced *C_s_* with *C_s_*-Γ and replaced *h_s_* with a function of *VPD*, as stomata respond more strongly to *VPD* than to *h*_s_ (Leuning, 1990). The BBL model is expressed as follows Equation 7:

(7) $$g_{SW}=m\frac{A}{\left(C_{s}-\Gamma )(1+\frac{VPD}{D_{0}}\right)}+g_{0}$$

Where Γ is the CO_2_ compensation point, μmol mol^−1^; *VPD* is the water vapor pressure difference, kPa; m, g_0_, and D_0_ are fitting parameters. According to Guo et al. (2022), the CO_2_ compensation points of C3 plants (Γ_C3_, μmol·mol^−1^) at various leaf temperatures (*T_L_*, °C) are given by the following formula following Equation 8:

(8) $$\Gamma_{C_{3}}=42.7+1.68(T_{L}-25)+0.012{\left(T_{L}-25\right)}^{2}$$

Where T_L_ is the leaf temperature by the photosynthesis meter, °C.

#### 2.4.3 USO model

The concept of optimal stomatal function suggests that stomata operate to enhance carbon uptake and reduce water loss. Their regulatory role is viewed as a constraint on photosynthesis during the regeneration of ribulose-1,5-bisphosphate. Under conditions where ambient CO_2_ levels substantially exceed Γ (Medlyn et al., 2012), the USO model (Medlyn et al., 2012) is shown in Equation 9, and g_1_ depends on the marginal water cost of plant carbon gain is shown in Equation 10.

(9) $$g_{SW}=1.6(1+\frac{g_{1}}{\sqrt{VPD}})\frac{A}{C_{s}}+g_{0}$$

(10) $$g_{1}=\sqrt{3\Gamma \lambda /1.6}$$

Where λ is the marginal water cost of leaf carbon gain, mol·mol^−1^; g_0_ and g_1_ are fitting parameters.

### 2.5 Model improvement

#### 2.5.1 Moisture response function

The formula for calculating the f(θ) is as follows Equation 11 (Keenan et al., 2010):

(11) $$f(\theta )=\{\begin{array}{cc} 0 & \theta <\theta_{w}\\ \frac{\theta -\theta_{w}}{\theta_{f}-\theta_{w}} & \theta_{w}\leq \theta \leq \theta_{f}\\ 1 & \theta >\theta_{f} \end{array}$$

Where *θ* is the soil weight moisture content, %; *θ_w_* and *θ_f_* are the wilting point and field capacity, %; $\frac{\theta -\theta_{w}}{\theta_{f}-\theta_{w}}$ is the relative moisture content of the soil.

Linear moisture response functions have been widely used to improve the BWB and BBL models under varied irrigation scenarios (Ji et al., 2017). In the USO model, λ represents the marginal water cost of leaf carbon gain (applicable under well-watered conditions where plant water status is not limiting) (Ji et al., 2017). Studies indicates that λ is predominantly influenced by plant functional type and soil moisture, exhibiting a positive correlation with soil moisture (Katul et al., 2012). Thus, by multiplying g_1_ by $\sqrt{f(\theta )}$to obtain an improved USO model as follows Equations 12–14.

(12) $$g_{SW}=m\frac{Ah_{s}f(\theta )}{C_{s}}+g_{0}$$

(13) $$g_{SW}=m\frac{A}{\left(C_{s}-\Gamma )(1+\frac{VPD}{D_{0}}\right)}f(\theta )+g_{0}$$

(14) $$g_{SW}=1.6(1+\frac{g_{1}\sqrt{f(\theta )}}{\sqrt{VPD}})\frac{A}{C_{a}}+g_{0}$$

#### 2.5.2 Leaf-air temperature differences

The ΔT serves as an indicator of the regulatory effects of soil moisture and plant water deficit on stomatal aperture (Soni et al., 2025). Thus, the following improvements were made to the *g*_sw_-models (Equation 15–17):

(15) $$g_{SW}=m\frac{Ah_{s}}{C_{s}}Exp(-\Delta T)+g_{0}$$

(16) $$g_{SW}=m\frac{A}{\left(C_{s}-\Gamma )(1+\frac{VPD}{D_{0}}\right)}Exp(-\Delta T)+g_{0}$$

(17) $$g_{SW}=1.6(1+\frac{g_{1}}{\sqrt{VPD}})\frac{A}{C_{a}}Exp(-\Delta T)+g_{0}$$

#### 2.5.3 Temperature response function

To account for temperature stress, the f(T) quantifies the constraining influence of temperature on plant development (Equation 18). The calculation method is as described by Yuan et al. (2007):

(18) $$f(T)=\frac{\left(T_{A}-T_{\min})(T_{A}-T_{\max}\right)}{[(T_{A}-T_{\min})(T_{A}-T_{\max})]-{\left(T_{A}-T_{op}\right)}^{2}}$$

Where *T_min_* and *T_max_* represent the minimum and maximum temperatures at which cotton can perform photosynthesis, set at 10°C and 40°C, respectively (Wang et al., 2016); *T_op_* is the optimal temperature for photosynthesis, at 30°C (Soni et al., 2025).

The following improvements were made to the BWB, BBL, and USO models (Equations 19–21):

(19) $$g_{SW}=m\frac{Ah_{s}f(T)}{C_{s}}+g_{0}$$

(20) $$g_{SW}=m\frac{A}{\left(C_{s}-\Gamma )(1+\frac{VPD}{D_{0}}\right)}f(T)+g_{0}$$

(21) $$g_{SW}=1.6(1+\frac{g_{1}\sqrt{f(T)}}{\sqrt{VPD}})\frac{A}{C_{a}}+g_{0}$$

#### 2.5.4 Interaction between water and temperature

Considering the dual influence of moisture and temperature on the stomatal conductance model, to determine which combination of stress factors is most effective in improving the model, improvements were performed using two-factor combinations [f(θ)&ΔT, f(T)&ΔT, f(θ)&f(T)] and multi-factor [f(θ)&ΔT&f(T)] combinations. The specific improvement equations for the *g*_sw_-models are as follows Equations 22–33:

①f(θ)&ΔT.

(22) $$g_{SW}=m\frac{Ah_{s}}{C_{s}}f(\theta )Exp(-\Delta T)+g_{0}$$

(23) $$g_{SW}=m\frac{A}{\left(C_{s}-\Gamma )(1+\frac{VPD}{D_{0}}\right)}f(\theta )Exp(-\Delta T)+g_{0}$$

(24) $$g_{SW}=1.6(1+\frac{g_{1}\sqrt{f(\theta )}}{\sqrt{VPD}})\frac{A}{C_{a}}Exp(-\Delta T)+g_{0}$$

②f(T)&ΔT.

(25) $$g_{SW}=m\frac{Ah_{s}}{C_{s}}f(T)Exp(-\Delta T)+g_{0}$$

(26) $$g_{SW}=m\frac{A}{\left(C_{s}-\Gamma )(1+\frac{VPD}{D_{0}}\right)}f(T)Exp(-\Delta T)+g_{0}$$

(27) $$g_{SW}=1.6(1+\frac{g_{1}\sqrt{f(T)}}{\sqrt{VPD}})\frac{A}{C_{a}}Exp(-\Delta T)+g_{0}$$

③f(θ)&f(T).

(28) $$g_{SW}=m\frac{Ah_{s}}{C_{s}}f(\theta )f(T)+g_{0}$$

(29) $$g_{SW}=m\frac{A}{\left(C_{s}-\Gamma )(1+\frac{VPD}{D_{0}}\right)}f(\theta )f(T)+g_{0}$$

(30) $$g_{SW}=1.6(1+\frac{g_{1}\sqrt{f(T)f(\theta )}}{\sqrt{VPD}})\frac{A}{C_{a}}+g_{0}$$

④f(θ)&ΔT&f(T).

(31) $$g_{SW}=m\frac{Ah_{s}}{C_{s}}f(\theta )f(T)Exp(-\Delta T)+g_{0}$$

(32) $$g_{SW}=m\frac{A}{\left(C_{s}-\Gamma )(1+\frac{VPD}{D_{0}}\right)}f(\theta )f(T)Exp(-\Delta T)+g_{0}$$

(33) $$g_{SW}=1.6(1+\frac{g_{1}\sqrt{f(T)f(\theta )}}{\sqrt{VPD}})\frac{A}{C_{a}}Exp(-\Delta T)+g_{0}$$

### 2.6 Model evaluation and statistical analysis

During calibration, the experimental observation data (n = 120) were fitted using nonlinear regression employing the’Dynamic Fit’module in Origin 2024 (OriginLab Corp., Northampton, MA, USA). Following the fitting process, model parameters, coefficients of determination (*R*², Equation 34), and statistical significance values were computed. Using 2023 data for calibration, followed by 2024 data for validation, the model was developed and tested. Model performance was evaluated based on root mean square error (RMSE, Equation 35), relative error (RE, Equation 36), improved efficiency coefficient (E_1_, Equation 37), and model slope (b_0_, Equation 38). The model goodness-of-fit indices were calculated as follows:

(34) $$R^{2}=\frac{{\left[\sum_{1}^{n}\left(O_{i}-\bar{O_{i}})(M_{i}-\bar{M_{i}}\right)\right]}^{2}}{\sum_{1}^{n}{\left(O_{i}-\bar{O_{i}}\right)}^{2}\sum_{1}^{n}{\left(M_{i}-\bar{M_{i}}\right)}^{2}}$$

(35) $$RMSE=\sqrt{\frac{\sum_{1}^{n}{\left(O_{i}-M_{i}\right)}^{2}}{n}}$$

(36) $$RE=\frac{\sum_{1}^{n}\left(\left|O_{i}-M_{i}\right|/O_{i}\right)}{n}$$

(37) $$E_{1}=1-\frac{\sum_{1}^{n}\left|O_{i}-M_{i}\right|}{\sum_{1}^{n}O_{i}-\bar{O_{i}}}$$

(38) $$b_{0}=\frac{\sum_{i=1}^{n}O_{i}M_{i}}{\sum_{i=1}^{n}O_{i}^{2}}$$

Where O_i_ represents the measured value, M_i_ is the estimated value, n is the sample size, and $\bar{O_{i}}$ and $\bar{M_{i}}$ are the average measured and estimated values, respectively. The smaller the RMSE and RE values, the better the simulation performance of the model. The higher the E_1_ value, the better is the simulation performance of the model. The slope parameter (b_0_) signifies the model’s systematic bias, where a value greater than 1 implies overestimation, and less than 1 reflects underestimation. A value nearer to 1 corresponds to improved simulation performance.

Statistical analysis of variance was conducted with IBM SPSS Statistics 22. Multiple mean comparisons were carried out with the least significant difference (LSD) test at a significance level of 0.05. Data visualization was performed using Origin 2024.

## 3 Results and analysis

### 3.1 Model parameter calculation

The fitting parameters and p values of the original BWB, BBL, and USO models and single-factor and multi-factor improved models are shown in **Table 3**. The g_sw_ regression fitting equations of the original BWB and BBL models were not significant under any treatment. However, the g_sw_ regression fitting equation of the original USO model was significant under all treatments, excluding F2B3 and CK treatments (p < 0.05) (**Table 3**). For the single-factor improved models (**Table 3**), the regression fitting equation of the USO model was significant under all treatments except CK treatment (p < 0.05). As ΔT improved, the regression fitting equations of the BWB, BBL, and USO models were significant (p < 0.05). For the multi-factor improved models (**Table 4**), the regression fitting equations of the BWB and BBL models under the joint improvement of f(θ) and f(T) were not significant, whereas those of the remaining BWB, BBL, and USO models were significant (p < 0.05). These findings indicate that g_sw_ is influenced by the factors incorporated in the g_sw_ model.

**Table 3:** Parameter estimation for the original, single-factor improved and multi-factor improved stomatal conductance model.

Model parameters			F1B1	F1B2	F1B3	F2B1	F2B2	F2B3	CK
O	BWB	m	6.71	14.54	11.05	18.00	16.14	8.45	1.74
		g_0_	92.71	86.34	90.19	64.48	88.04	115.62	111.04
		p	ns	ns	ns	ns	ns	ns	ns
	BBL	m	56.73	191.91	287.80	216.89	263.08	217.63	11.45
		D_0_	-5.27×10^11^	-2.2×10^11^	2.35×10^11^	-6.2×10^9^	-1.2×10^9^	-5.6×10^12^	-1.0×10^9^
		g_0_	113.37	95.51	95.51	107.26	120.32	121.47	117.87
		p	ns	ns	ns	ns	ns	ns	ns
	USO	g_1_	278.59	578.42	503.77	612.87	546.61	471.24	73.45
		g_0_	87.90	78.56	77.70	67.88	89.56	99.25	109.62
		p	*	**	*	**	*	ns	ns
S	BWB-f(θ)	m	16.81	22.26	18.83	29.53	23.52	32.73	1.87
		g_0_	84.22	87.91	90.26	67.77	90.99	58.06	113.5
		p	ns	ns	ns	ns	ns	*	ns
	BBL-f(θ)	m	137.88	310.12	538.26	418.79	385.48	839.17	21.12
		D_0_	-379.08	3.96×10^11^	-7895.91	1.04×10^11^	1.10×10^10^	-3002.67	-1222.1
		g_0_	110.99	117.64	92.1	103.87	121.64	74.75	117.32
		p	ns	ns	*	ns	ns	ns	ns
	USO-f(θ)	g_1_	445.23	730.46	683.4	776.46	650.29	890.51	107.23
		g_0_	172.86	78.24	75.79	70.07	91.75	69.73	108.15
		p	*	*	*	**	*	**	ns
	BWB-ΔT	m	5.66	7.27	8.7	9.99	12.13	7.6	4.12
		g_0_	92.21	92.15	79.77	77.27	60.63	94.08	94.86
		p	*	*	*	*	*	*	*
	BBL-ΔT	m	157.23	214.88	217.15	323.38	362.4	220.16	30.88
		D_0_	-7322.5	-886.16	-2760.03	-149.8	-743.65	-8686	-146.06
		g_0_	92.83	93.39	86.82	78	74.94	98.27	114.89
		p	*	*	*	*	*	*	*
	USO-ΔT	g_1_	269.44	515.97	414.97	577.55	459.79	273.58	54.91
		g_0_	91.09	92.88	93.48	77.6	105.98	123.4	112.6
		p	*	*	*	**	**	**	**
	BWB-f(T)	m	6.58	12.28	8.78	16.54	13.74	4.88	1.1
		g_0_	96.83	105.88	106.98	81.14	108.91	133.19	114.83
		p	ns	ns	ns	ns	ns	ns	ns
	BBL-f(T)	m	52.08	156.64	241.01	231.29	227.47	88.29	9.61
		D_0_	1.31×10^12^	-1486.17	8.22×10^10^	-383.4	-136.08	-1072.56	-321
		g_0_	115.19	128.02	109.66	110.81	131.45	139.34	118.33
		p	ns	ns	ns	ns	ns	ns	ns
	USO-f(T)	g_1_	632.59	767.28	684.17	898.89	625.24	889.69	60.53
		g_0_	84.53	95.63	94.96	82.51	110.84	90.9	114.4
		p	<0.05	<0.05	<0.05	<0.01	<0.05	<0.05	ns
M	BWB-f(θ)&ΔT	m	10.14	11.22	13.72	15.31	18.36	7.76	6.1
		g_0_	93.75	92.96	83.36	81.65	61.15	101.65	95.15
		p	*	***	***	***	***	***	*
	BBL-f(θ)&ΔT	m	281.61	329.22	332.8	487.24	469.85	216.72	88.92
		D_0_	-4423.8	-384.13	-1232.2	-283.55	-141.48	9.33×10^11^	-4878.3
		g_0_	94.19	94.67	90.96	82.98	78.78	106.27	109.34
		p	*	***	***	***	***	***	*
	USO-f(θ)&ΔT	g_1_	199.75	260.95	313.83	361.49	397.71	260.52	149.33
		g_0_	92.01	90.19	77.38	75.38	63.71	95.68	94.33
		p	***	***	***	***	***	***	***
	BWB-f(T)&ΔT	m	6.19	7.25	10.53	10.35	13.01	7.55	3.3
		g_0_	93.9	104.63	88.57	84.6	76.94	108.64	103.48
		p	*	*	*	*	*	*	*
	BBL- f(T)&ΔT	m	177.87	244.62	341.99	348.63	483.46	247.24	24.31
		D_0_	-2790.36	-3434	3.21×10^10^	-1022.9	-7251.8	-953.37	-216.91
		g_0_	93.77	102.2	86.17	84.65	72.22	111.42	116.81
		p	*	*	*	**	**	*	ns
	USO- f(T)&ΔT	g_1_	269.67	324.56	395.24	448.45	486.71	261.88	196.13
		g_0_	92.55	90.62	79.24	77.51	64.45	99.66	92.74
		p	***	***	***	***	***	***	***
	BWB-f(θ)&f(T)	m	15.89	17.82	13.48	26.98	19.24	19.03	0.04
		g_0_	90.65	108.77	109.63	83.67	112.61	108.17	118.94
		p	ns	ns	ns	ns	ns	ns	ns
	BBL-f(θ)&f(T)	m	124.54	239.01	420.68	414.79	304.34	595.62	13.18
		D_0_	-1003.32	-6945.57	-1943.04	1.74×10^10^	-175.49	-4161.2	-933.04
		g_0_	113.41	128.61	109.31	109.65	134.26	110.64	118.2
		p	ns	ns	ns	ns	ns	ns	ns
	USO- f(θ)&f(T)	g_1_	209.11	267.12	394.31	369.41	457.92	263.24	130.39
		g_0_	92.83	96.68	76.22	79.38	67.1	104.43	99.92
		p	*	**	***	***	***	***	***
	BWB- f(θ)&ΔT&f(T)	m	11.16	11.28	18.89	15.84	20.89	8.05	5.08
		g_0_	95.16	105.07	86.63	88.45	75.16	112.88	103
		p	*	*	*	*	*	*	*
	BBL- f(θ)&ΔT&f(T)	m	319.05	380.38	591.22	524.16	748.76	254.76	72.8
		D_0_	1.07×10^12^	-208.44	2.97×10^10^	-108	-1090.8	-561.35	-2211.9
		g_0_	95.03	102.78	84.79	89.14	71.11	114.91	113.16
		p	*	*	**	**	**	*	ns
	USO- f(θ)&ΔT&f(T)	g_1_	283.19	333.39	519.19	458.56	571.21	269.34	174.67
		g_0_	93.26	96.93	75.92	81.33	66.21	107.17	98.3
		p	***	***	***	***	***	***	*

O denotes the original stomatal conductance model, S denotes the single-factor improved stomatal conductance model, and M denotes the multi-factor improved stomatal conductance model. * indicates p < 0.05, ** indicates p < 0.01, *** indicates p < 0.001, and ns indicates that the simulated curve is not significant.

**Table 4:** Evaluation results of the original, single-factor improved and multi-factor improved stomatal conductance model.

Model parameters			2023							2024						
			F1B1	F1B2	F1B3	F2B1	F2B2	F2B3	CK	F1B1	F1B2	F1B3	F2B1	F2B2	F2B3	CK
O	BWB	*R* ^2^	0.06	0.12	0.12	0.13	0.08	0.03	0.01	0.03	0.09	0.1	0.05	0.08	0.02	0.02
		RMSE	59.47	107.46	12.34	20.9	23.23	15.6	12.76	49.71	44.44	13.14	11.14	16.13	15.16	7.24
		RE	0.29	0.49	0.01	0.03	0.02	0.01	0.01	0.86	0.55	0.05	0.03	0.05	0.05	0.02
		E_1_	-0.2	-0.13	-0.07	0.01	-0.08	-0.07	-0.01	-1.13	-2.43	-1.9	-1.56	-3.77	-2.76	-1.54
		b_0_	0.76	0.67	0.77	0.62	0.6	0.72	0.68	1.38	1.43	1.44	1.39	1.63	1.53	1.37
	BBL	*R* ^2^	0.01	0.04	0.13	0.03	0.02	0.02	0.01	0.05	0.14	0.08	0.05	0.02	0.17	0.01
		RMSE	58.51	108.84	12.45	22.12	23.84	15.68	12.61	55.45	51.72	12.81	11.61	16.85	15.03	7.68
		RE	0.27	0.43	0.01	0.03	0.01	0.01	0.01	0.98	0.66	0.05	0.04	0.06	0.05	0.02
		E_1_	-0.18	-0.08	-0.09	0.04	-0.02	-0.05	0.02	-1.38	-2.99	-1.77	-1.93	-4.04	-2.69	-1.69
		b_0_	0.74	0.64	0.77	0.57	0.58	0.72	0.67	1.45	1.53	1.43	1.48	1.72	1.54	1.39
	USO	*R* ^2^	0.12	0.02	0.01	0.07	0.02	0.01	0.01	0.09	0.19	0.09	0.08	0.08	0.15	0.09
		RMSE	59.5	104.18	11.88	20.67	23.13	15.53	12.76	46.99	42.45	12.09	11.23	14.48	13.78	7.08
		RE	0.29	0.46	0.01	0.03	0.01	0.01	0.01	0.81	0.51	0.04	0.03	0.05	0.05	0.02
		E_1_	-0.18	-0.09	-0.03	0.03	-0.04	-0.07	-0.02	-1	-2.16	-1.52	-1.4	-3.29	-2.32	-1.48
		b_0_	0.77	0.68	0.79	0.62	0.6	0.74	0.68	1.35	1.41	1.36	1.39	1.6	1.48	1.36
S	BWB-f(θ)	*R* ^2^	0.1	0.11	0.12	0.15	0.06	0.2	0.08	0.04	0.08	0.11	0.14	0.09	0.25	0.04
		RMSE	58.4	105.93	12.37	20.64	23.3	14.39	12.73	48.71	47.04	13.29	11.38	16.58	10.69	7.48
		RE	0.29	0.46	0.01	0.04	0.01	0.01	0.01	0.82	0.58	0.05	0.03	0.05	0.03	0.02
		E_1_	-0.17	-0.11	-0.09	0.06	-0.06	0.01	0	-1.07	-2.61	-1.98	-1.5	-3.91	-1.43	-1.64
		b_0_	0.77	0.66	0.76	0.62	0.6	0.8	0.67	1.36	1.46	1.46	1.38	1.67	1.25	1.38
	BBL-f(θ)	*R* ^2^	0.02	0.04	0.14	0.05	0.02	0.19	0.07	0.01	0.13	0.11	0.04	0.07	0.16	0.01
		RMSE	58.46	108.85	12.4	22	23.84	14.96	12.64	54.75	51.88	12.56	11.39	17.02	9.67	7.66
		RE	0.28	0.43	0.01	0.03	0.01	0.01	0.01	0.97	0.66	0.05	0.04	0.06	0.03	0.02
		E_1_	-0.17	-0.07	-0.1	0.05	-0.01	0.04	0.02	-1.35	-3.02	-1.75	-1.82	-4.09	-1.25	-1.69
		b_0_	0.74	0.64	0.77	0.58	0.57	0.79	0.67	1.45	1.54	1.42	1.46	1.74	1.28	1.39
	USO-f(θ)	*R* ^2^	0.12	0.15	0.2	0.15	0.08	0.17	0.02	0.1	0.17	0.13	0.16	0.11	0.14	0.1
		RMSE	86.46	104.17	11.92	20.54	23.17	14.89	12.76	130.12	43.57	11.82	11.44	14.82	11.58	7.11
		RE	0.62	0.46	0.01	0.04	0.01	0.01	0.01	2.24	0.51	0.04	0.03	0.05	0.03	0.02
		E_1_	-1	-0.08	-0.04	0.06	-0.03	-0.02	-0.02	-4.89	-2.24	-1.51	-1.38	-3.38	-1.55	-1.5
		b_0_	1.31	0.68	0.79	0.63	0.6	0.77	0.68	2.44	1.42	1.36	1.39	1.62	1.34	1.36
	BWB-ΔT	*R* ^2^	0.1	0.27	0.34	0.4	0.4	0.43	0.11	0.25	0.38	0.37	0.42	0.45	0.43	0.21
		RMSE	53.43	88.75	9.66	16.69	14.43	11.04	11.65	44.64	35.55	10	8.14	8.88	9.86	6.06
		RE	0.26	0.24	0.01	0	0.01	0.01	0.01	0.78	0.45	0.04	0.02	0.02	0.03	0.01
		E_1_	-0.07	0.31	0.16	0.32	0.37	0.32	0.14	-0.92	-1.71	-1.16	-1	-1.33	-1.45	-1.04
		b_0_	0.8	0.77	0.85	0.75	0.85	0.86	0.74	1.35	1.34	1.24	1.31	1.19	1.15	1.29
	BBL-ΔT	*R* ^2^	0.26	0.33	0.26	0.33	0.4	0.43	0.11	0.35	0.3	0.43	0.4	0.46	0.46	0.06
		RMSE	53.36	93.5	10.22	17.58	18.29	12.02	12.18	43.56	33.22	9.88	8.36	10.32	9.61	7.61
		RE	0.26	0.26	0.01	0	0.01	0.01	0.01	0.76	0.41	0.04	0.02	0.03	0.03	0.02
		E_1_	-0.07	0.27	0.12	0.33	0.23	0.29	0.05	-0.82	-1.44	-1.11	-0.94	-1.72	-1.34	-1.66
		b_0_	0.8	0.75	0.84	0.78	0.83	0.84	0.68	1.34	1.32	1.26	1.29	1.17	1.14	1.39
	USO-ΔT	*R* ^2^	0.12	0.3	0.43	0.43	0.42	0.56	0.12	0.19	0.29	0.24	0.42	0.44	0.54	0.22
		RMSE	54.13	129.21	10.96	15.73	14.43	11.36	11.64	47.02	38.08	14.34	13.31	10.04	9.22	7.37
		RE	0.27	0.45	0.01	0	0.01	0.01	0.01	0.81	0.72	0.05	0.04	0.06	0.06	0.02
		E_1_	-0.09	-0.22	0.06	0.14	0.18	0.24	0.1	-1.01	-3.52	-2.12	-2.12	-1.4	-1.33	-1.59
		b_0_	0.88	1.17	1.11	1.05	1.08	1.01	0.71	1.33	1.33	1.27	1.29	1.15	1.12	1.38
	BWB-f(T)	*R* ^2^	0.19	0.01	0.07	0.13	0.07	0.01	0.02	0.04	0.11	0.01	0.07	0.05	0.03	0.04
		RMSE	59.59	106.12	12.44	20.9	23.18	15.38	12.74	49.99	52.27	14.29	11.43	16.91	16.97	7.43
		RE	0.29	0.46	0.01	0.04	0.01	0.01	0.01	0.87	0.67	0.06	0.03	0.06	0.06	0.02
		E_1_	-0.21	-0.11	-0.07	0.03	-0.06	0	-0.01	-1.15	-3.08	-2.21	-1.76	-4.14	-3.18	-1.61
		b_0_	0.76	0.65	0.74	0.61	0.6	0.71	0.67	1.38	1.54	1.52	1.44	1.71	1.61	1.38
	BBL-f(T)	*R* ^2^	0.18	0.01	0.06	0.04	0.02	0.1	0.01	0.01	0.18	0	0.06	0.03	0.16	0.01
		RMSE	58.41	108.33	12.55	22.03	23.72	15.38	12.62	55.84	57.11	14.1	11.74	17.63	17.29	7.69
		RE	0.27	0.42	0.01	0.03	0.01	0.01	0.01	0.99	0.73	0.05	0.04	0.06	0.06	0.02
		E_1_	-0.18	-0.06	-0.09	0.03	-0.02	0.01	0.02	-1.4	-3.43	-2.12	-1.99	-4.32	-3.28	-1.7
		b_0_	0.74	0.62	0.74	0.57	0.57	0.71	0.67	1.46	1.6	1.51	1.49	1.76	1.62	1.4
	USO-f(T)	*R* ^2^	0.12	0.1	0.12	0.14	0.05	0.15	0.01	0.09	0.19	0.15	0.08	0.08	0.18	0.1
		RMSE	72.36	105.88	12.55	20.64	22.65	16.62	12.59	65.82	68.11	17.69	18.36	19.78	18.62	7.63
		RE	0.44	0.57	0.01	0.06	0.02	0.02	0.01	1.1	0.83	0.07	0.05	0.06	0.06	0.02
		E_1_	-0.42	-0.15	-0.09	-0.07	-0.09	-0.21	-0.01	-1.85	-4.17	-2.81	-3.16	-4.9	-3.29	-1.7
		b_0_	1.03	0.81	0.94	0.81	0.68	0.84	0.69	1.62	1.73	1.67	1.77	1.87	1.65	1.4
M	BWB-f(θ)&ΔT	*R* ^2^	0.15	0.26	0.34	0.37	0.41	0.42	0.17	0.26	0.37	0.44	0.34	0.42	0.52	0.21
		RMSE	52.1	89.55	9.74	17.12	15.05	11.15	11.4	45.75	36.64	10.68	8.31	9.32	10.11	6.47
		RE	0.25	0.24	0.01	0	0.01	0.01	0.01	0.8	0.46	0.04	0.02	0.03	0.03	0.01
		E_1_	-0.04	0.3	0.17	0.32	0.35	0.34	0.15	-0.96	-1.81	-1.35	-0.98	-1.43	-1.52	-1.2
		b_0_	0.8	0.76	0.84	0.74	84	0.86	0.73	1.37	1.35	1.34	1.31	1.28	1.31	1.32
	BBL-f(θ)&ΔT	*R* ^2^	0.15	0.28	0.26	0.3	0.42	0.52	0.15	0.35	0.32	0.41	0.38	0.42	0.5	0.05
		RMSE	51.92	94.09	10.29	18.02	18.85	12.11	11.79	44.42	34.13	10.46	8.35	9.88	10.25	7.49
		RE	0.25	0.26	0.01	0	0.01	0.01	0.01	0.77	0.43	0.04	0.02	0.03	0.04	0.02
		E_1_	-0.04	0.26	0.14	0.31	0.25	0.33	0.1	-0.86	-1.55	-1.3	-0.94	-1.66	-1.52	-1.57
		b_0_	0.8	0.75	0.84	0.72	0.79	0.87	0.7	1.36	1.32	1.34	1.32	1.37	1.34	1.38
	USO-f(θ)&ΔT	*R* ^2^	0.15	0.39	0.42	0.42	0.54	0.6	0.17	0.27	0.41	0.55	0.48	0.62	0.62	0.23
		RMSE	55.92	90.88	10.33	17.44	16.69	11.45	11.65	40.49	29.71	7.89	6.62	6.21	8.78	5.49
		RE	0.27	0.21	0.01	0.01	0.01	0.01	0.01	0.71	0.38	0.03	0.02	0.02	0.03	0.01
		E_1_	-0.16	0.34	0.13	0.3	0.32	0.34	0.13	-0.69	-1.24	-0.65	-0.41	-0.55	-1.16	-0.83
		b_0_	0.73	0.7	0.77	0.66	0.81	0.88	0.7	1.3	1.28	1.2	1.2	1.16	1.28	1.26
	BWB-f(T)&ΔT	*R* ^2^	0.09	0.24	0.24	0.39	0.5	0.34	0.05	0.23	0.31	0.35	0.43	0.52	0.41	0.12
		RMSE	53.76	92.07	10.57	17.01	14.8	11.94	12.19	45.16	43.53	11.45	8.83	10.76	12.58	6.58
		RE	0.26	0.27	0.01	0	0.01	0.01	0.01	0.79	0.56	0.04	0.03	0.03	0.04	0.02
		E_1_	-0.09	0.22	0.11	0.27	0.32	0.28	0.09	-0.94	-2.36	-1.55	-1.16	-2.13	-2.12	-1.28
		b_0_	0.8	0.73	0.82	0.74	0.82	0.82	0.71	1.36	1.44	1.39	1.35	1.38	1.43	1.33
	BBL- f(T)&ΔT	*R* ^2^	0.19	0.17	0.2	0.33	0.49	0.48	0.06	0.25	0.28	0.37	0.39	0.55	0.47	0.04
		RMSE	53.67	95.27	10.63	17.73	16.47	12.43	12.37	43.52	39.97	10.71	8.86	10.39	12.76	7.67
		RE	0.27	0.29	0.01	0	0.01	0.01	0.01	0.76	0.51	0.04	0.03	0.03	0.04	0.02
		E_1_	-0.1	0.19	0.11	0.27	0.27	0.26	0.03	-0.83	-2.04	-1.27	-1.11	-1.77	-2.13	-1.69
		b_0_	0.8	0.73	0.83	0.72	0.8	0.82	0.67	1.34	1.41	1.36	1.36	1.38	1.46	1.4
	USO- f(T)&ΔT	*R* ^2^	0.11	0.28	0.39	0.42	0.61	0.54	0.07	0.28	0.4	0.53	0.47	0.6	0.51	0.25
		RMSE	53.53	90.93	9.15	16.59	14.44	12.09	12.44	47.09	36.93	10.03	9.85	8.75	10.29	5.95
		RE	0.26	0.27	0.01	0	0.01	0.01	0.01	0.82	0.47	0.04	0.03	0.02	0.03	0.01
		E_1_	-0.07	0.21	0.19	0.29	0.33	0.29	0.1	-1.01	-1.8	-1.14	-1.22	-1.3	-1.52	-1
		b_0_	0.87	0.81	0.88	0.84	0.86	0.88	0.77	1.43	1.38	1.34	1.35	1.3	1.3	1.3
	BWB-f(θ)&f(T)	*R* ^2^	0.09	0.07	0.05	0.13	0.05	0.09	0.05	0.04	0.08	0	0.04	0.02	0.02	0.05
		RMSE	58.84	106.35	12.55	20.76	23.27	15.05	12.67	49.07	54.57	14.41	11.68	17.39	14.89	7.67
		RE	0.29	0.45	0.01	0.04	0.01	0.01	0.01	0.85	0.69	0.06	0.03	0.06	0.05	0.02
		E_1_	-0.18	-0.09	-0.08	0.06	-0.05	-0.01	0.02	-1.1	-3.25	-2.29	-1.73	-4.28	-2.69	-1.69
		b_0_	0.76	0.64	0.74	0.61	0.59	0.74	0.67	1.37	1.56	1.54	1.43	1.74	1.51	1.39
	BBL-f(θ)&f(T)	*R* ^2^	0.02	0.02	0.06	0.04	0.01	0.07	0.01	0.01	0.15	0.01	0.05	0.08	0.15	0.02
		RMSE	58.41	108.32	12.63	21.94	23.73	15.35	12.66	55.25	57.66	13.95	11.67	17.9	14.42	7.67
		RE	0.27	0.41	0.01	0.04	0.01	0.01	0.01	0.98	0.74	0.05	0.04	0.06	0.05	0.02
		E_1_	-0.17	-0.05	-0.1	0.05	-0.01	-0.02	0.02	-1.37	-3.47	-2.14	-1.94	-4.4	-2.52	-1.69
		b_0_	0.74	0.62	0.74	0.57	0.57	0.74	0.67	1.45	1.61	1.51	1.48	1.78	1.51	1.39
	USO- f(θ)&f(T)	*R* ^2^	0.03	0.05	0.08	0.09	0.12	0.15	0.03	0.11	0.14	0.01	0.05	0.12	0.13	0.1
		RMSE	67.5	119.3	16.46	23.95	27.96	17.11	13.66	40.32	30.71	7.38	6.26	5.71	9.8	5.77
		RE	0.33	0.35	0.01	0.01	0.01	0.01	0.01	0.69	0.39	0.02	0.02	0.01	0.03	0.01
		E_1_	-0.46	-0.08	-0.37	-0.05	-0.15	0	-0.09	-0.63	-1.34	-0.41	-0.34	-0.41	-1.44	-0.96
		b_0_	0.64	0.5	0.56	0.44	0.37	0.6	0.62	1.24	1.27	1.11	1.12	1.08	1.3	1.27
	BWB- f(θ)&ΔT&f(T)	*R* ^2^	0.14	0.23	0.26	0.35	0.52	0.53	0.09	0.25	0.3	0.36	0.4	0.5	0.51	0.13
		RMSE	52.43	92.71	10.48	17.45	14.99	12	11.99	46.21	44.41	11.54	9.02	11.47	12.41	6.85
		RE	0.25	0.27	0.01	0	0.01	0.01	0.01	0.8	0.57	0.04	0.03	0.03	0.04	0.05
		E_1_	-0.07	0.22	0.12	0.26	0.31	0.3	0.1	-0.98	-2.45	-1.61	-1.17	-2.25	-2.09	-1.39
		b_0_	0.8	0.73	0.82	0.73	0.82	0.82	0.71	1.37	1.45	1.4	1.35	1.22	1.22	1.35
	BBL- f(θ)&ΔT&f(T)	*R* ^2^	0.14	0.23	0.27	0.39	0.57	0.56	0.09	0.27	0.28	0.37	0.35	0.53	0.47	0.07
		RMSE	52.22	95.74	10.65	18.19	16.76	12.65	12.18	44.39	40.53	10.48	8.97	10.91	12.41	7.58
		RE	0.25	0.29	0.01	0	0.01	0.01	0.01	0.78	0.52	0.04	0.03	0.03	0.04	0.06
		E_1_	-0.07	0.19	0.1	0.27	0.27	0.25	0.06	-0.87	-2.09	-1.28	-1.12	-1.8	-2.04	-1.65
		b_0_	0.8	0.72	0.83	0.71	0.83	0.83	0.68	1.35	1.41	1.36	1.36	1.21	1.23	1.39
	USO- f(θ)&ΔT&f(T)	*R* ^2^	0.14	0.27	0.4	0.41	0.6	0.65	0.1	0.27	0.37	0.52	0.45	0.63	0.64	0.18
		RMSE	52.39	89.4	9.32	16.62	14.57	11.82	11.79	44.18	38.76	9.33	8.74	8.95	11.48	6.28
		RE	0.25	0.25	0.01	0	0.01	0.01	0.01	0.77	0.49	0.03	0.02	0.02	0.04	0.01
		E_1_	-0.05	0.27	0.18	0.31	0.33	0.31	0.12	-0.87	-1.95	-1.01	-0.95	-1.34	-1.82	-1.16
		b_0_	0.8	0.76	0.82	0.75	0.84	0.84	0.73	1.37	1.39	1.3	1.33	1.21	1.2	1.32

O denotes the original stomatal conductance model, S denotes the single-factor improved stomatal conductance model, and M denotes the multi-factor improved stomatal conductance model.

### 3.2 Model evaluation results and comparison

The evaluation index results for the original *g*_sw_-models, single-factor and multi-factor improved models, are presented in **Table 4**.

#### 3.2.1 Evaluation of the original model

**Figure 2:**
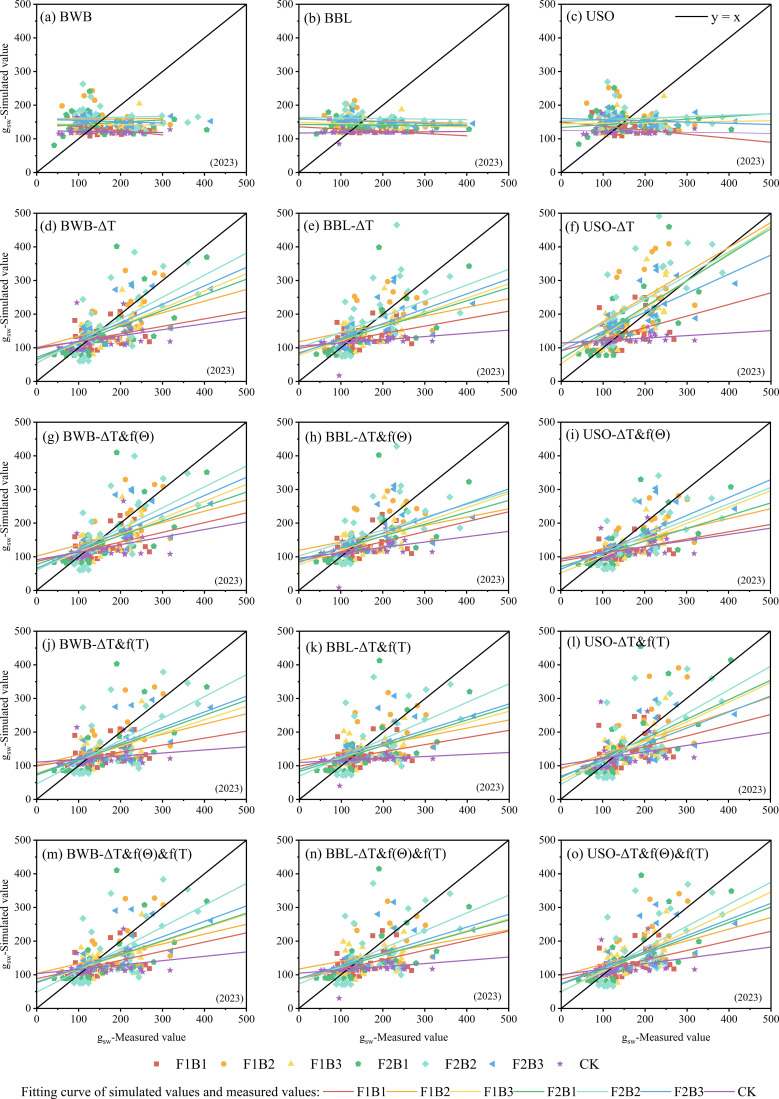
**(a–o)** Linear comparison of simulated values and experimental values for the original and improved stomatal conductivity models (2023).

**Figure 3:**
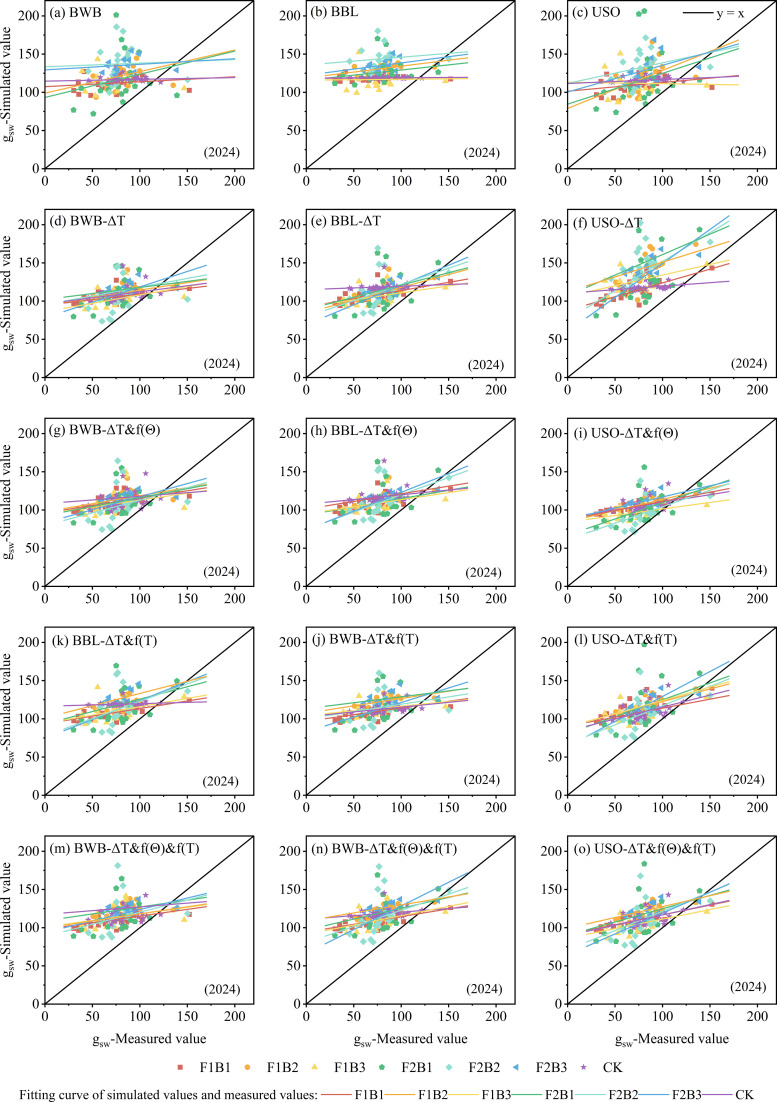
**(a–o)** Linear comparison of simulated values and experimental values for the original and improved stomatal conductivity models (2024).

The three original models, BWB, BBL, and USO, showed poor performance in simulating g_sw_ in cotton leaves and were unable to accurately characterize the dynamic patterns of g_sw_ in cotton leaves (**Figures 2**, **3a–c**). Under all treatments in both 2023 and 2024, the R^2^ values for all three models remained extremely low (<0.15). Under a majority of the treatments, the R^2^ value was <0.10, indicating that the original models had extremely weak explanatory power regarding the variability in cotton leaf g_sw_ and showed severe model fit deficiency. The root mean square error (RMSE) values for all three models were usually high. In 2023, the RMSE value was >20 under a majority of the treatments, with that under F1B2 treatment exceeding 100. In 2024, although the RMSE value decreased under some treatments, it remained high, indicating a significant difference between the simulated and measured values and suggesting that simulation accuracy failed to meet the study requirements. The RE value significantly deviated from zero, and E1 was predominantly negative. These results indicated that the original models not only failed to accurately capture the trends in g_sw_ but also exhibited systematic bias, resulting in extremely low reliability of the simulation results. Furthermore, no clear pattern in simulation performance was observed between different irrigation treatments (F1B1–F2B3) and CK treatment, indicating that the original models do not fully account for the regulatory effects of field water and fertilizer management on cotton g_sw_. The model structure and parameters were incompatible with the actual cultivation conditions of cotton fields in Xinjiang; therefore, improving simulation performance through parameter optimization or model refinement is necessary.

#### 3.2.2 Evaluation of the single-factor improved model

Compared with the original models (R^2^ < 0.15), the BWB, BBL, and USO models with single-factor modification showed improved simulation performance for cotton leaf g_sw_. However, significant differences were observed in the optimization results among the models. The optimized model incorporating ΔT showed significant improvement (**Figures 2**, **3d–f**). Its R^2^ value increased to >0.4 for a majority of the treatments, its RMSE value substantially decreased compared to that of the original model, and its RE approached 0. On the contrary, the models incorporating only f(θ) or f(T) exhibited poor fitting performance (R^2^ < 0.2), indicating that ΔT is the key single-factor for improving the simulation accuracy of cotton g_sw_ models. This factor can effectively compensate for the structural deficiencies of the original model and significantly enhance the model’s ability to simulate the dynamic changes in g_sw_ under different irrigation conditions in cotton fields in Xinjiang. However, models incorporating a single-factor have the limitation of insufficient universality; consequently, it is necessary to couple multiple factors to enhance the simulation performance of the model.

#### 3.2.3 Evaluation of the multi-factor improved model

After multi-factor combinations were introduced, the simulation performance of the improved BWB, BBL, and USO models showed significant improvement. In particular, the models incorporating the ΔT&f(θ), ΔT&f(T), and ΔT&f(θ) &f(T) combinations yielded excellent results (*R*^2^ > 0.5), whereas the f(θ)–f(T) combination showed poor results (*R*^2^ < 0.15). Linear comparison plots of simulated and measured values were plotted for the combinations that yielded better simulation results (ΔT&f[θ], ΔT&f[T], and ΔT&f[θ]&f[T]) (**Figures 2**, **3g–o**). Based on the R^2^, RMSE, RE, E1, and b_0_ values, the USO model showed the highest simulation accuracy, followed by the BBL model, whereas the BWB model showed relatively poor performance. Furthermore, the simulation accuracy achieved by introducing the ΔT&f(θ) &f(T) combination was superior to that achieved by introducing the ΔT&f(θ) and ΔT&f(T) combinations, with the *R*^2^ value exceeding 0.6. Among the BWB, BBL, and USO models incorporating the ΔT&f(θ) &f(T) combination, the 2-year *R*^2^ value of the USO model improved by 3.77%–49.75% and 1.94%–55.98% compared to those of the BBL and BWB models, respectively, and by 0.35%–38.10% and 8.99%–45.21%, respectively.

The simulated g_sw_ values under F2B2 and F2B3 treatments, which had higher lower limits for irrigation, were closer to the observed g_sw_ values than those under other treatments. Among the BWB, BBL, and USO models incorporating the ΔT&f(θ)&f(T) combination, the 2-year *R*^2^ and b_0_ values under F2B2 treatment improved by 46.42%–518.23% and 0.27%–22.10%, respectively, and by 26.24%–631.53% and 6.87%–16.01%, respectively, compared to those under other treatments, whereas the RE value decreased by 0.45%–96.26% and 2.74%–96.78%, respectively. Under F2B3 treatment, the 2-year *R*^2^ and b_0_ values increased by 51.13%–524.68% and 28.25%–553.17%, respectively, compared to those under other treatments, whereas the RE value decreased by 16.60%–97.06% and 0.43%–22.10%. The closer the R^2^ and b_0_ values are to 1, the smaller the RE value and the better the model simulation performance. These findings indicate that treatments with higher lower irrigation limits yield higher simulation accuracy in the g_sw_ model. Therefore, ΔT combined with f(θ), f(T), or f(θ)&f(T) should be incorporated into the USO and BBL models to simulate g_sw_. This approach yields more accurate simulations, particularly for treatments with higher irrigation thresholds during the squaring, flowering, and boll setting stages of cotton.

### 3.3 Model applicability evaluation

**Figure 4:**
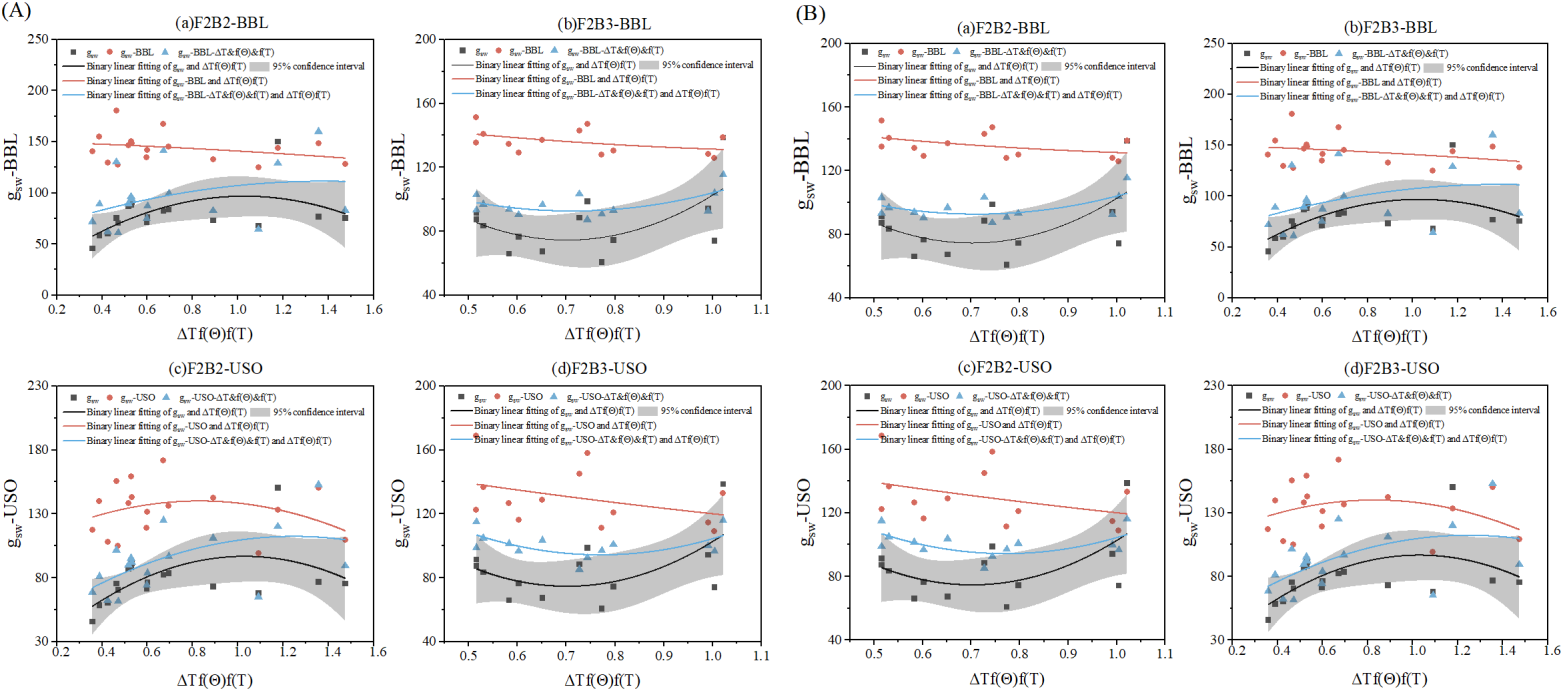
**(A, B)** Comparison of fitting curves for F2B2 and F2B3 processing using the combined BBL and USO models with improved ΔT&f(θ)&f(T).

The improved model with the best simulation accuracy (BBL combined with USO and ΔT&f[θ]&f[T]) was selected for applicability evaluation under F2B2 and F2B3 treatments. The evaluation results are shown in **Figure 4**. After a comparison of the 95% confidence intervals (CIs) of fitting curves between the original model (g_sw_), the improved model (g_sw_-ΔT&f[θ]&f[T]), and the measured g_sw_ values, the appropriate improvement factor (ΔT&f[θ]&f[T]) range for the BBL and USO models was determined.

In 2023 (**Figure 4A**), under F2B2 and F2B3 treatments, when ΔT&f(θ)&f(T) was within the [0, 1] interval, the improved BBL and USO models reduced the original simulated values and slightly overestimated the measured g_sw_ values. When ΔT–f(θ)–f(T) was within the [1, 6] and [1, 4] intervals, the improved BBL and USO models increased the original simulated values but underestimated the measured g_sw_ values. When ΔT&f(θ)&f(T) was within the [6, 7.5] and [4, 6.5] intervals, the improved models increased the original simulated values but overestimated the measured g_sw_ values. In 2024 (**Figure 4B**), the improved models reduced the original simulated values but overestimated the measured g_sw_ values. Under F2B2 and F2B3 treatments, the simulated values were closer to the measured g_sw_ values when ΔT&f(θ)&f(T) was within the [0.8, 1] and [0.6, 1] intervals. These results indicate that the improved models, particularly the USO model, are more applicable to the F2B3 treatment with a high irrigation lower limit. The simulated curves of the improved BBL and USO models under F2B3 treatment fell within the 95% CI of the observed data and exhibited a stronger agreement with the actual measurements. Therefore, incorporating the ΔT&f(θ)&f(T) combination improves the applicability of the g_sw_ model, and this improved model is more stable under different f(θ), ΔT, and f(T) conditions.

## 4 Discussion

In this study, soil moisture content in cotton fields was manipulated by adjusting the upper and lower limits of irrigation, thereby influencing the stomatal conductance of cotton leaves. Three traditional models, BWB, BBL, and USO were used for g_sw_ simulation. Results revealed that the simulation accuracy of all three models was low. The USO model showed the best performance, followed by the BBL model, whereas the BWB model performed the worst. Given the growth environment and physiological characteristics of cotton under plastic film drip irrigation in this study, and from the perspective of the theoretical foundations of g_sw_ models (Gardner et al., 2022), the core reason for the performance differences among the models can be explained as follows: the USO model includes only one stomatal regulation parameter (g_1_), and the setting of this parameter aligns more closely with the fundamental physiological mechanisms of stomatal movement (El-Sharkawy et al., 1985). The dynamic regulation of g_sw_ is essentially a simplified response of plants to environmental signals. A single key parameter can reduce the accumulation of errors caused by multi-parameter coupling, thereby improving simulation stability (Ji et al., 2017); On the contrary, the BWB and BBL models incorporate multiple environmental response parameters, whose parameter settings are more suitable for crop types in non-arid regions (Büker et al., 2007; Uddling and Pleijel, 2006). The soil water–thermal heterogeneity caused by subsurface drip irrigation, combined with the specific regulatory patterns of cotton stomata, consequently leads to significant simulation deviations (Li et al., 2024a). This is closely related to the scenario-specific nature of g_sw_ models. The parameter systems of different models are constructed based on specific crops and environments. When the application scenario changes, the mismatch between the parameters and the actual physiological processes becomes immediately apparent.

To improve model fit, this study incorporated f(θ), ΔT, and f(T) to refine the three models. All modifications improved simulation accuracy, with ΔT incorporation yielding the best results among single-factor improvements (HÉRoult et al., 2012). Combined multi-factor improvements (ΔT & f(θ), ΔT & f(T), and ΔT & f(θ) & f(T)) produced even better results, with R² values approaching 0.6 or higher. ΔT is a direct indicator of soil moisture and plant water deficit (Zhang et al., 2019). Changes in ΔT can rapidly trigger feedback regulation of cotton stomata. An increase in ΔT (leaf temperature exceeds air temperature) indicates that the transpiration rate of plants exceeds the water uptake rate of roots. Under these conditions, stomata close to reduce water loss while simultaneously limiting CO_2_ uptake. This process is achieved through changes in turgor pressure within the guard cells. The inclusion of ΔT enables the precise capture of this dynamic regulatory process (Medlyn et al., 2012). f(θ) quantifies the regulation of g_sw_ by soil moisture content, essentially reflecting the balance between root water uptake capacity and stomatal transpiration demand. When soil moisture is insufficient, restricted root water uptake is transmitted to the leaves through xylem sap signals (e.g., increased abscisic acid [ABA] content), inhibiting the water uptake and turgor of guard cells and consequently decreasing g_sw_. f(T) accounts for the effects of temperature on stomatal enzyme activity and guard cell physiology and metabolism. At optimal temperatures, guard cell metabolism is active and g_sw_ is at its peak; however, too high or too low temperatures inhibit metabolic processes, leading to a decline in stomatal regulatory capacity. The interactions among multiple factors primarily result in synergistic regulatory effects (Wang et al., 2019). Changes in ΔT are often directly associated with soil moisture content and ambient temperature. Insufficient soil moisture leads to increased plant transpiration, which in turn increases ΔT. High-temperature environments further amplify this effect, causing rapid stomatal closure (Hofmann et al., 2025). Conversely, adequate soil moisture alleviates the transpiration stress caused by high temperatures, reduces ΔT, and maintains normal stomatal opening (Jiang et al., 2020). This interaction makes it difficult for single-factor improvements to fully capture the dynamic changes in g_sw_. However, multi-factor improvements can cover the multidimensional physiological processes of stomatal regulation, thereby significantly enhancing simulation accuracy. Li et al. (2023) improved the simulation accuracy of a g_sw_ model for mulched corn by introducing ΔT and f(θ). Building on this foundation, this study incorporated f(T) into g_sw_ models, addressed the specific characteristics of cotton under plastic-mulched drip irrigation in the arid regions of Northern Xinjiang, and achieved multi-factor synergistic improvements, thereby filling a gap in existing research regarding multi-factor improvements for cotton, which is a major cash crop, under mulched conditions in arid regions (Wang et al., 2024). Compared with previous studies focused on improving g_sw_ models for food crops such as wheat and maize, this study focused on the specific response of cotton stomata to drought stress. The improvement process used in this study is better aligned with the cultivation characteristics of economic crops and clarifies the central role of ΔT in simulating crop g_sw_ in arid regions (Li et al., 2024b). This complements the water-factor-dominated conclusions drawn by a majority of the studies on crop g_sw_ in arid regions, further refining the theoretical framework for simulating g_sw_ for mulched crops under arid conditions.

Different levels of water deficit exert significant inhibitory effects on cotton g_sw_, with the inhibitory effect increasing as the deficit becomes more severe (Li et al., 2025; Zou et al., 2022). Herein, F2B2 and F2B3 treatments (upper limit of high irrigation) yielded the best simulation results. A high irrigation lower limit can maintain a stable and suitable soil moisture environment, placing cotton plants in a state of mild water stress. Under these conditions, the water uptake capacity of the root system and the transpiration demand of leaves are in dynamic equilibrium and the turgor pressure of guard cells remains stable. Changes in g_sw_ are primarily regulated by environmental factors (light, temperature, and humidity), exhibiting greater regularity and better alignment with the parameter settings and physiological assumptions of the improved model (Aroca et al., 2017). However, under severe water stress, cotton plants activate emergency regulatory mechanisms, leading to the massive accumulation of stress hormones such as ABA. This causes the stomata to remain in a semi-closed state, and changes in g_sw_ no longer follow conventional environmental response patterns. Furthermore, the regulatory roles of factors such as ΔT and f(θ) are dominated by hormonal regulation, resulting in increased simulation errors (Lin et al., 2015). This finding is consistent with previous findings indicating that plant stomatal regulation exhibits strong regularity under mild water stress, leading to higher simulation accuracy (Sweet et al., 2017). However, this study further clarifies the optimizing effect of the upper limit of high irrigation rates on the simulation of cotton g_sw_ under plastic-mulched drip irrigation conditions, thereby supplementing the applicable conditions for the cotton g_sw_ model in the Changji region (Han et al., 2024).

This study has several limitations. First, there are limitations in the experimental design. The study was performed at a single location in Northern Xinjiang using a single cotton variety, without considering the difference in stomatal physiology among different cotton varieties (e.g., stomatal density and guard cell size) or the climatic and soil heterogeneity across different regions (e.g., the various oases in Northern Xinjiang and the cotton-growing areas in Southern Xinjiang). Consequently, the generalizability of the findings to other regions and their applicability to different varieties are limited. Second, there are limitations in model refinement. This study incorporated only environmental factors (soil moisture, temperature, and leaf–air temperature difference) for model improvement, without addressing the intrinsic physiological factors of cotton plants (e.g., leaf ABA content, chlorophyll content, and stomatal density). Consequently, the model’s physiological mechanism remains relatively weak and cannot fully explain the physiological processes involved in g_sw_ regulation. Third, there are limitations in the validation scope. The improved model was validated solely using field trial data from this study, and independent trial data from other regions or years were not included. Therefore, the stability and reliability of the improved model require further verification. Given these limitations, future studies should focus on three specific aspects. First, the scope of experiments should be expanded by establishing trial sites in other cotton-growing regions of Xinjiang and selecting cotton varieties of different genotypes to verify the regional applicability and varietal adaptability of the improved model while simultaneously collecting multi-year data to enhance the temporal stability of the model. Second, the understanding of the physiological mechanisms of the model should be enhanced by incorporating cotton leaf physiological factors (e.g., ABA content, stomatal density, and photosynthetic rate) to construct a model coupling environmental factors, physiological factors, and g_sw_. This approach will further refine the model’s physiological foundation and improve its simulation accuracy. Third, the practical application of the improved g_sw_ model should be promoted by coupling it with cotton growth models (e.g., the WOFOST model) to achieve accurate predictions of cotton photosynthetic rates and yields at the regional scale, thereby providing more scientific theoretical support for water management of cotton under plastic-mulched drip irrigation in Changji.

## 5 Conclusions

Through simulation verification and applicability evaluation of three stomatal conductivity models (BWB, BBL, and USO), and their single-factor (f(θ), ΔT, f(T)) and multi-factor (f(θ)&ΔT, f(θ)&f(T), f(T)&ΔT, f(θ)&ΔT&f(T)) modified variants, this study found that the USO model exhibited the highest simulation accuracy, followed by the BBL and BWB models. The introduction of multi-factor combinations such as ΔT&f(θ), ΔT&f(T), or ΔT&f(θ)&f(T) significantly improved simulation accuracy, indicating that ΔT is a key factor in enhancing the simulation accuracy of cotton stomatal conductance. For cotton grown under plastic-mulched drip irrigation in Changji under the experimental conditions of this study, it is recommended to adopt the USO model incorporating the multi-factor combination of ΔT&f(θ)&f(T). This multi-factor-enhanced model is more suitable for irrigation environments where the lower limit of soil moisture is maintained at approximately 70% of field capacity over a long period (F2B2 and F2B3 treatments). In addition, this model can be validated and extended to cotton-growing regions with similar climate, soil, and cultivation practices to those in this study.

## Data Availability

The original contributions presented in the study are included in the article/**Supplementary Material**. Further inquiries can be directed to the corresponding authors.
