# Supplementary material for: Improved construction and applicability analysis of stomatal conductance model for cotton under drip irrigation in Northern Xinjiang
Source: Front Plant Sci. 2026 May 11;17:1827847. doi: 10.3389/fpls.2026.1827847 (PMC13199926; doi:10.3389/fpls.2026.1827847)
Supplement: Supplementary file 2 [file SupplementaryFile2.docx]

Appendix Table 1 Physical properties of test soil

| Soil layer/cm | Particle proportion/% | | | Soil bulk density/(g·cm^-3^) | Field capacity/% | Soil texture |
| --- | --- | --- | --- | --- | --- | --- |
|  | ＜0.002 mm | 0.002~0.02mm | 0.02～0.2 mm |  |  |  |
| 0~10 | 15.6 | 44.4 | 38.4 | 1.52 | 27 | clay loam |
| 10~20 | 13.5 | 47.9 | 37.4 | 1.59 | 26 | Sandy loam |
| 20~30 | 12.3 | 46.3 | 40.1 | 1.49 | 26 | Sandy loam |
| 30~40 | 15.6 | 48.1 | 35.4 | 1.45 | 27 | Sandy loam |
| 40~50 | 15.3 | 33.4 | 48.2 | 1.48 | 27 | clay loam |
| 50~60 | 14.8 | 46.6 | 36.6 | 1.57 | 37 | Sandy loam |
| 60~70 | 25.7 | 44.3 | 28.8 | 1.45 | 29 | loamy clay |
| 70~80 | 14.7 | 40.9 | 44.2 | 1.44 | 32 | loam |
| 80~90 | 11.0 | 36.4 | 51.8 | 1.44 | 28 | loam |
| 90~100 | 11.6 | 37.2 | 50.6 | 1.40 | 31 | loam |

Appendix Table 2 Explanation of symbols and their units

| Symbol | Explanation of Symbols | Unit |
| --- | --- | --- |
| *T_L_* | Leaf temperature by the photosynthesis meter | ℃ |
| *h_s_* | Relative humidity by the photosynthesis meter | % |
| A | Net photosynthetic rate by the photosynthesis meter | μmol⋅m^−2^⋅s^−1^ |
| *g_sw_* | Stomatal conductance | mol⋅m^−2^⋅s^−1^ |
| *C_i_* | Intercellular carbon dioxide concentration by the photosynthesis meter | μmol⋅mol^−1^ |
| *C_s_* | The CO_2_ concentration on the leaf surface by the photosynthesis meter | μmol⋅mol^−1^ |
| S | Daily solar radiation by the weather station | MJ⋅m^−2^ |
| *RH* | Relative humidity by the weather station | % |
| *T_a_* | Air temperature by the weather station | ℃ |
| *SVP* | Saturated vapor pressure | kPa |
| *AVP* | Actual vapor pressure | kPa |
| *T_dew_* | Dew point temperature | ℃ |
| Γ | The CO_2_ compensation point (Γ_C3_) in BBL model | μmol⋅mol^−1^ |
| λ | Marginal water cost of leaf carbon gain in USO model | mol⋅mol^−1^ |
| *θ* | Soil weight moisture content | % |
| *θ_w_* | Wilting point | % |
| *θ_f_* | Field capacity | % |
| f(θ) | Moisture response function | — |
| ΔT | Leaf-air temperature differences | ℃ |
| *T_min_* | Minimum temperatures at which cotton can perform photosynthesis (10℃) | ℃ |
| *T_max_* | Maximum temperatures at which cotton can perform photosynthesis (40℃) | ℃ |
| *T_op_* | Optimal temperature for photosynthesis (30℃) | ℃ |
| f(T) | Temperature response function | — |
